# Feasibility of a noninvasive heart failure telemonitoring system: A mixed methods study

**DOI:** 10.1177/20552076241272633

**Published:** 2024-09-12

**Authors:** Teemu Ekola, Vesa Virtanen, Tuomas H Koskela

**Affiliations:** 1 Faculty of Medicine and Health Technology, Tampere University, Tampere, Finland; 2 The Wellbeing Services County of Pirkanmaa, Finland; 3 Heart Hospital, Tampere University Hospital, Tampere, Finland

**Keywords:** Heart failure, telemonitoring, telemetry, remote patient monitoring, telemedicine, feasibility study, mixed methods

## Abstract

**Objective:**

The aim of this study was to examine the feasibility of a noninvasive telemonitoring system used by heart failure patients and nurses in a pilot program of the Heart Hospital unit in Tampere, Finland.

**Methods:**

This cross-sectional observational study used a mixed methods design. Quantitative data were collected with one self-generated questionnaire for patients, and qualitative data were collected with a questionnaire for patients and semi-structured focus group interviews for patients and nurses. The questionnaire was sent to 47 patients who were in the pilot program, and 29 patients (61.7%) responded. Purposefully selected 8 patients and 8 nurses attended the interviews. We used descriptive statistics to assess the quantitative data from the questionnaire and inductive thematic analysis to identify themes deriving from the focus group interviews. We categorized the themes into facilitators and barriers to telemonitoring.

**Results:**

Both the quantitative and qualitative data show that the telemonitoring system is easy to use, supports self-care and self-monitoring, and increases the feeling of safety. The chat tool of the system facilitated communication. The patients and nurses considered the system reliable despite some technical problems. The focus group interviews addressed technical challenges, nurses’ increased workload, and patients’ engagement with daily follow-up as possible barriers to telemonitoring.

**Conclusions:**

The noninvasive heart failure telemonitoring system used in the pilot program is feasible. We found facilitators and barriers to telemonitoring that should be considered when developing the noninvasive telemonitoring of heart failure in the future.

## Introduction

Heart failure is a global health problem with an increasing prevalence and burden to patients and healthcare systems.^1–3^ The prevalence of heart failure is about 1–2% in adults, but over 10% in those aged 70 or over.^1,3^ The prognosis of heart failure has improved because of the development of treatments, but it is still poor, especially in the older population.^1,4^ Additionally, the number of heart failure hospitalizations has increased in recent decades and is expected to increase even further due to population growth, aging, and the increasing prevalence of comorbidities.^1,5^ Frequent long hospitalizations result in substantial burdens and expenses to healthcare systems and are associated with an increased risk of mortality.^3^

The aging population and increasing number of hospitalizations have created a need for new care models that could reduce heart failure patients’ hospitalizations. Due to digitalization and advanced telecommunication technologies, there has been a growing interest in implementing and studying telemedicine and telemonitoring in the heart failure population. Telemedicine and telemonitoring enable patients to provide remote digital health information to support and optimize their care.^1,6^ Self-care can be enhanced by improving patients’ knowledge and adherence through distance education and telemonitoring.^1,6^ Enhanced patient self-care and more intensive monitoring might help to prevent or identify exacerbations of heart failure earlier and avoid hospitalizations.^7^

Telemonitoring can mean structured telephone support, noninvasive (e.g. a scale or blood pressure meter) or invasive (e.g. a pacemaker or pulmonary artery sensor) devices that allow health data and other useful information to be sent between patients and healthcare professionals, or a range of wearable technologies such as patches, watches, or textiles that can monitor and collect health data.^7^ According to the guidelines of the European Society of Cardiology (ESC) in 2021, noninvasive telemonitoring may be considered for patients with heart failure to reduce the risk of recurrent hospitalizations and mortality, but further evidence on invasive systems is required.^1^

The previous meta-analyses for the noninvasive telemonitoring of heart failure have reported that telemonitoring reduces all-cause mortality and heart failure-related hospitalizations compared to usual care.^6,8–11^ However, there have also been some conflicting results.^11–17^ The adherence of patients to telemonitoring has mostly been high, and telemonitoring has improved their self-care.^6,14,18,19^ Over a longer follow-up time, the adherence reduces, however.^20^ Many studies report increased quality of life for patients with telemonitoring,^6,17,19^ but there are also some conflicting results.^21^

Despite the rising number of noninvasive telemonitoring programs and studies for heart failure patients globally, only a few studies have assessed the feasibility of using telemonitoring systems.^20,22–27^ The methods most used in these studies include questionnaires, interviews, or a combination of the two.^20,22,23,25–27^ Two studies have used the theoretical UTAUT (unified theory of acceptance and use of technology) model or its extension, UTAUT2, in their methodology.^20,23^ Feasibility is an important factor regarding patients’ and healthcare professionals’ adherence to telemonitoring systems and the success of the implementation of telemonitoring programs.^20,22^ Therefore, it is necessary to obtain more information about the feasibility of noninvasive heart failure telemonitoring systems.

Noninvasive telemonitoring for heart failure patients has previously been very scarce in Finland, but lately, many pilots with various telemonitoring systems have begun in different parts of the country. In August 2020, the Heart Hospital in Pirkanmaa Hospital District in Finland deployed a pilot program with a noninvasive heart failure telemonitoring system produced by Siemens Healthcare Ltd. To the best of our knowledge, the feasibility of this telemonitoring system has not been studied before.

The aim of this study was to examine the feasibility of the noninvasive telemonitoring system used by heart failure patients and nurses in the pilot program of the Heart Hospital unit in Tampere, Finland.

## Methods

### Study design

This is a cross-sectional observational study. We used a mixed methods design and combined both quantitative and qualitative methods to examine the feasibility of the telemonitoring system. We collected data with questionnaires and interviews to assess end-users’ views from both patients and nurses using the telemonitoring system.

### Study population

At the time of the study, the telemonitoring system was used in Heart Hospital units in Tampere and Hämeenlinna and in one health center of Tampere city. Approximately, 50 patients were using the telemonitoring system in the pilot program at that time. All patients who attended the telemonitoring program at the Heart Hospital unit in Tampere (47 patients) and all professionals using the telemonitoring system (8 nurses) were asked to participate in the study.

Patients were recruited in the telemonitoring program from the cardiology outpatient clinics and the inpatient wards of the Heart Hospital unit in Tampere according to the inclusion criteria. The inclusion criteria were an age of at least 18 years, hospitalization for heart failure within the last 18 months, and NYHA-class II–III. The exclusion criteria included a referral made for heart transplant assessment, the patient waiting for heart transplantation or already having a heart or vascular transplant, end-stage kidney dysfunction, and conditions or diseases that might prevent or substantially weaken the ability to use the telemonitoring system (e.g. dementia, schizophrenia, known drug addiction, difficult sense deficit, being under guardianship, or living in continuous controlled institutional care). No division into a study group and a control group was made.

### Telemonitoring system

The telemonitoring system in this study consists of an Android-based tablet computer and a digital scale. The touch-screen tablet computer includes a web browser, YouTube, and the “Digital Hands” application by Siemens Healthcare Ltd. The application allows daily automatic questionnaires and different measurements of patient health as well as communication and the sharing of care-associated information between the patient and nurses. The tablet is connected to a scale via Bluetooth and uses a GSM signal to transmit data to the Digital Hands cloud service located in Norway.

With the telemonitoring system, the patient answers six questions about his/her health condition via the application and measures his/her weight daily. The questions used in the application are in Textbox 1. The patient answers every question with a yes or no. The answers are scored automatically: if the patient answers no, there comes 0 points, but if he/she answers yes, there come 3 points for the medication question and 1 point for other questions.

Textbox 1. Daily questions in the application.

1. Has your condition weakened since yesterday?
2. Do you have increasing shortness of breath?
3. Does the shortness of breath hamper you lying down?
4. Have you noticed changes in your heart rhythm?
5. Have you noticed increased swelling in your stomach or legs?
6. Have you taken your medication as prescribed?

**Figure 1.**
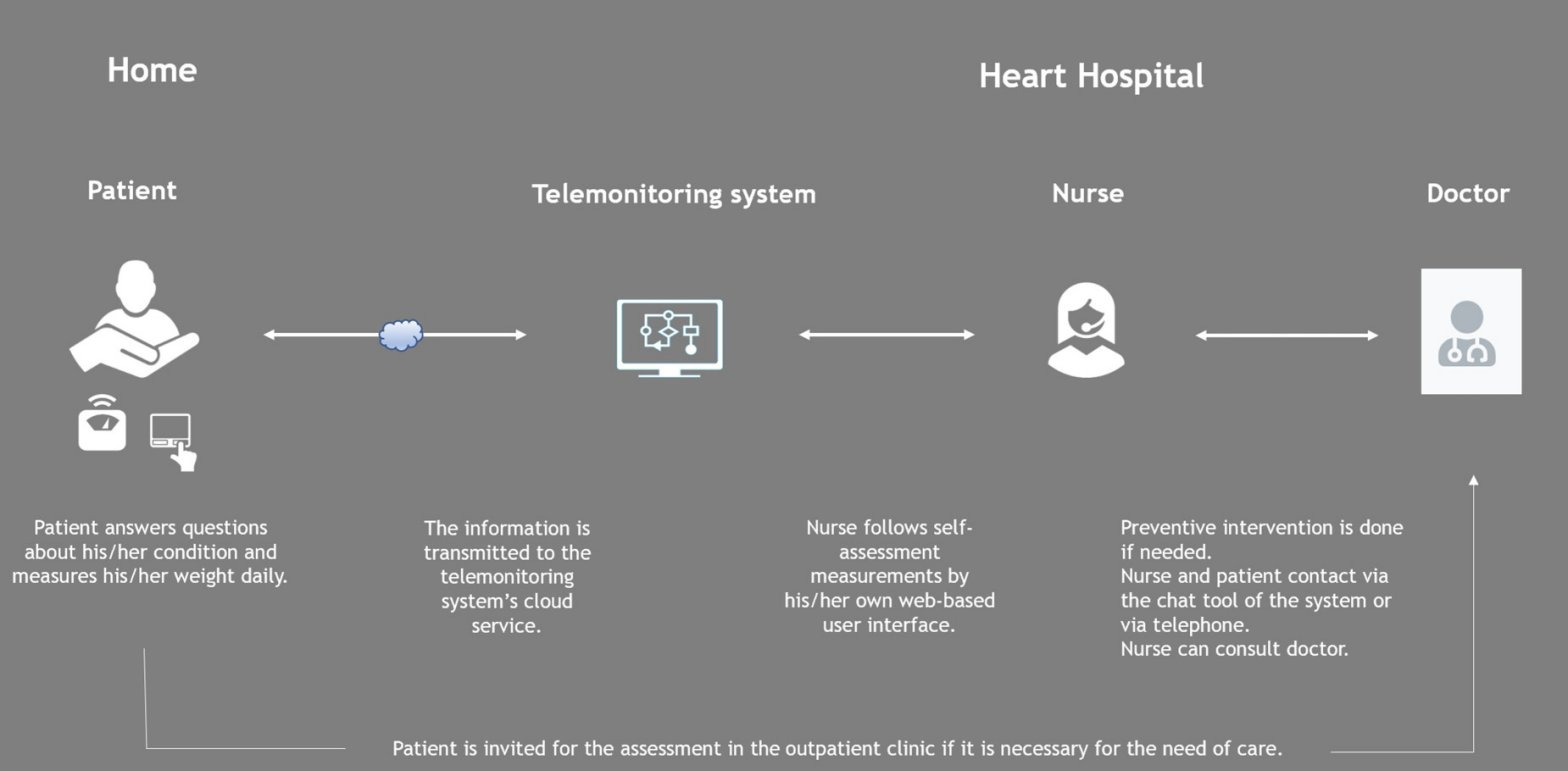
Telemonitoring process in the Heart Hospital.

After answering, the application sends the information to the cloud service of the system and an automatic self-care feedback message to the patient. Nurses who are involved in the patient's care can see the patient's self-assessment measurements through their web-based user interface. The nurses follow the patient's measurements during working hours on weekdays. If the patient's score is 3 or more or the weight change is over the alarm limits, nurses contact patients via the application's chat tool or telephone for an intervention. The intervention can include patient guidance or change in the medication, doctor consultation, reservation for a phone appointment with a doctor, or an invitation for the assessment in the heart failure outpatient clinic. If the patient has alarming symptoms (difficulty to breathe, chest pain, serious arrhythmia, or weak general condition), he/she is instructed to call the emergency number. The intervention is recorded in the medical records system. The objective is to detect possible exacerbations of heart failure early and avoid hospitalization. If the patient sends a message via the chat tool, the nurses reply the next business day at the latest. The whole process of telemonitoring is shown in Figure 1. The images of the application and the user interface for nurses are found in Appendix 1.

### Data collection

**Figure 2.**
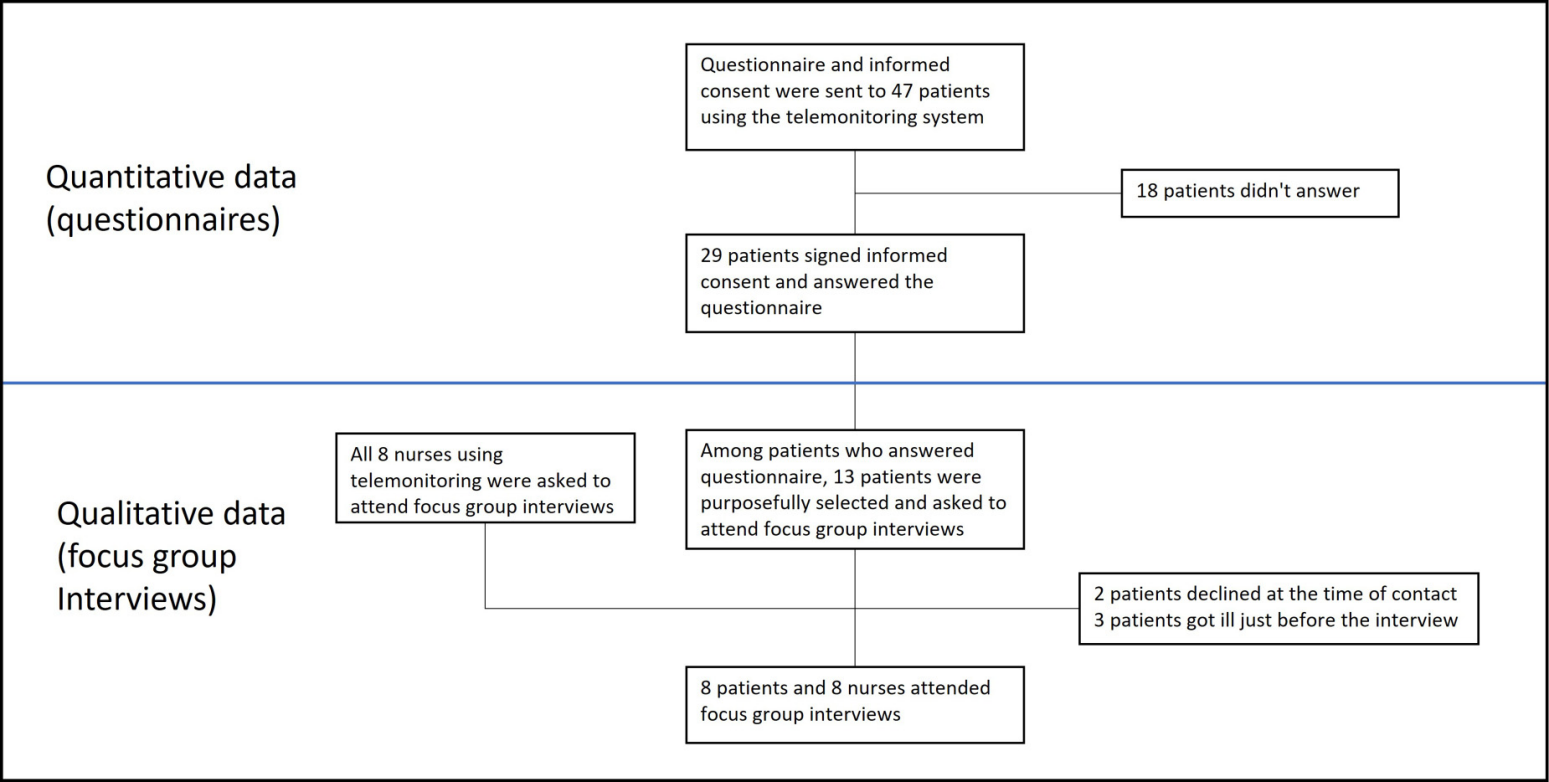
Data collection flow.

The data collection of the study was based on one questionnaire for patients and semi-structured focus group interviews with nurses and patients. Quantitative data were collected by using the questionnaire, and the qualitative data were collected with the questionnaire and focus group interviews. The data collection flow of the study is shown in Figure 2.

#### Quantitative data

We used one self-generated questionnaire to assess the end-user experiences and demographics of the patients. There was no completely applicable validated questionnaire for this purpose, so we had to plan our own. The clarity and comprehensibility of the questionnaire were pilot-tested with one heart failure patient. The collected demographic characteristics included age, gender, height, weight, time with heart failure, cardiovascular comorbidities, treatments for heart failure, and former use of digital devices. The questionnaire assessed the feasibility of the telemonitoring system using a Likert scale (from 1 meaning “complete agreement” to 5 meaning “complete disagreement”). We used statements about the reliability and ease of using the scale, tablet, and application as well as the relevance, comprehensiveness, and comprehensibility of the content in the application. We also asked how telemonitoring had affected the patient's life generally. We used a net promoter score to assess how likely patients would recommend the telemonitoring system to a friend or relative. The questionnaire is included in its entirety in Appendix 2.

The data was collected at a single timepoint. The questionnaire and the informed consent declaration form of the study were sent by post in December 2021 to all patients attending noninvasive heart failure telemonitoring at the Heart Hospital unit in Tampere (47 patients). A reminder was sent in March 2022 to those patients who had not answered the first time. In the end, 29 patients (61.7%) answered the questionnaire and signed the informed consent form.

#### Qualitative data

Semi-structured focus group interviews were conducted to obtain more detailed information about the user experiences of patients and nurses using the telemonitoring system.

We carried out five interviews between February 2022 and May 2022, three for patients and two for nurses. For the interviews, we purposefully selected patients from all patients who had answered the questionnaire and signed the informed consent form. We invited patients of varying ages, digital skills, and answers in the questionnaire to achieve more discussion and different opinions in the interviews. In addition, we invited all nurses using the telemonitoring system to interviews. Patients were contacted by telephone and nurses by email.

In the end, eight patients and eight nurses attended the interviews. Due to last-minute absences, two patient interviews were conducted with three patients and one interview with two patients. One nurse interview was with five nurses and the other with three nurses. The nurses and patients were interviewed separately because their questions varied to some extent.

All interviews were conducted face-to-face in a meeting room of the Heart Hospital unit in Tampere. Due to the long travel distance, one nurse attended the interview via remote connection. All nurses signed an informed consent form before the beginning of their interviews. The male interviewer (TE) is an MD and an employee of one health center in Tampere. He conducted the interviews as part of his PhD studies on heart failure telemonitoring. He had used the telemonitoring system before and was a colleague of two of the interviewed nurses. He was instructed to organize focus group interviews by an associate professor of general practice at Tampere University (TK), who is experienced in research and conducting focus group interviews. Only the interviewer and the invited patients or nurses were present in the interviews.

The frameworks for the interviews and the interview questions were planned in the fall of 2021 by the research group. They were modified by the answers to the questionnaire and the gathered experience between the interviews. The eventual frame for the patient interview is included in Appendix 3, and the eventual frame for the nurse interview is included in Appendix 4.

The nurses and patients were aware of the goals for the interview and TE's reasons for conducting the interviews. The topics for the interviews and questions were provided by the interviewer. The interviews were audio-recorded with a recorder and a smartphone. An official service provider transcribed the recordings verbatim in the summer of 2022. The recordings were disposed of securely after transcription, and the answers were anonymized in the transcribed versions. The transcripts were not returned to the participants. No field notes were made. The interviews took from 38 to 95 min (38, 74, 85, 86, and 95 min). In the last interview, the data seemed to be saturated as new issues regarding our research question did not appear in the discussion. The last interview also took the shortest time.

In addition, the questionnaire for patients had some open-ended questions about general comments, development ideas, and technical challenges with the telemonitoring system.

### Data analysis

#### Quantitative data

We used descriptive statistics to assess the quantitative data from the questionnaires. We analyzed the data with IBM SPSS Statistics Version 28.

The quantitative data was reported according to the STROBE checklist for cross-sectional observational studies, as applicable. The STROBE checklist is included in Appendix 5.

#### Qualitative data

We analyzed the transcribed interviews and used inductive thematic analysis to identify the codes and themes that derived from the data.

First, the transcripts were read by the authors (TE, VV, and TK) separately. The authors searched for meaningful sentences and coded them in Excel. Then, they examined them and collated the most frequent and relevant items together. Thereafter, they separately clustered the codes into themes. These steps were done first for the patient interview transcripts and then for the nurse interview transcripts. Then all authors compared and discussed the codes and themes together and in consensus categorized them into facilitators and barriers to telemonitoring. From the interview transcripts, the authors also identified factors affecting the patient selection for telemonitoring and development ideas for telemonitoring. Finally, TE named and translated these themes, factors, and development ideas from Finnish to English, and the names were validated by the other authors (TK and VV). All authors are MDs. TK is an associate professor of general practice at Tampere University and VV is a docent of cardiology and a part-time employee and a former chief physician of the Heart Hospital.

The qualitative data was reported according to the COREQ criteria.^28^ The COREQ checklist is included in Appendix 6.

Responses to the open-ended questions of the questionnaire were analyzed by the author TE.

## Results

The characteristics of the participants are presented in Table 1. The nurses had used the telemonitoring system for 0.5–1.5 years with a mean of 1 year. Six nurses were from Heart Hospital units and two nurses were from one health center of Tampere city. The demographics of the eight patients who attended the focus group interviews varied slightly from all the patients who had answered the questionnaire. Of those interviewed patients, five patients (62.5%) were female, and four patients (50%) had had heart failure for 3–9 years. The other demographics of the patients attending the focus group interviews were similar to those of all the patients answering the questionnaire.

**Table 1.** Work experience of nurses participating in the focus group interviews (*n* = 8) and characteristics of patients answering the questionnaire (*n* = 29).

	Range	*N* (valid %)	Mean	SD
**Nurses**		8		
**Work experience in healthcare (years)**	5–26		14.4	7.09
< 10		2 (28.6)		
10–20		4 (57.1)		
> 20		1 (14.3)		
**Patients**		29		
**Age (years)**	27–86		61.4	12.14
≤ 49		3 (10.3)		
50–59		9 (31)		
60–69		9 (31)		
≥ 70		8 (27.6)		
**Gender**				
Male		22 (75.9)		
Female		7 (24.1)		
**BMI**	20.47–48.00		31.02	7.59
**Time with heart failure (years)**				
0–2		12 (42.9)		
3–9		9 (32.1)		
≥ 10		7 (25)		
**Cardiovascular comorbidities**				
Coronary artery disease		5 (17.2)		
Hypertension		20 (69)		
Valvular heart disease		7 (24.1)		
Atrial arrhythmia		12 (41.4)		
Cardiomyopathy		17 (58.6)		
**Treatments**				
PCI^a^		2 (6.9)		
Pacemaker		18 (62.1)		
Medication		28 (96.6)		
Other (e.g. CABG^b^, TAVI^c^, or rehabilitation)		4 (13.8)		
**Earlier use of digital devices (e.g. computer,** **tablet computer, or mobile phone)**				
			
Daily		26 (89.7)		
Weekly		2 (6.9)		
Monthly		1 (3.4)		

^a^PCI: percutaneous coronary intervention.

^b^CABG: coronary artery bypass graft surgery.

^c^TAVI: transcatheter aortic valve implantation.

### Quantitative data

**Figure 3.**
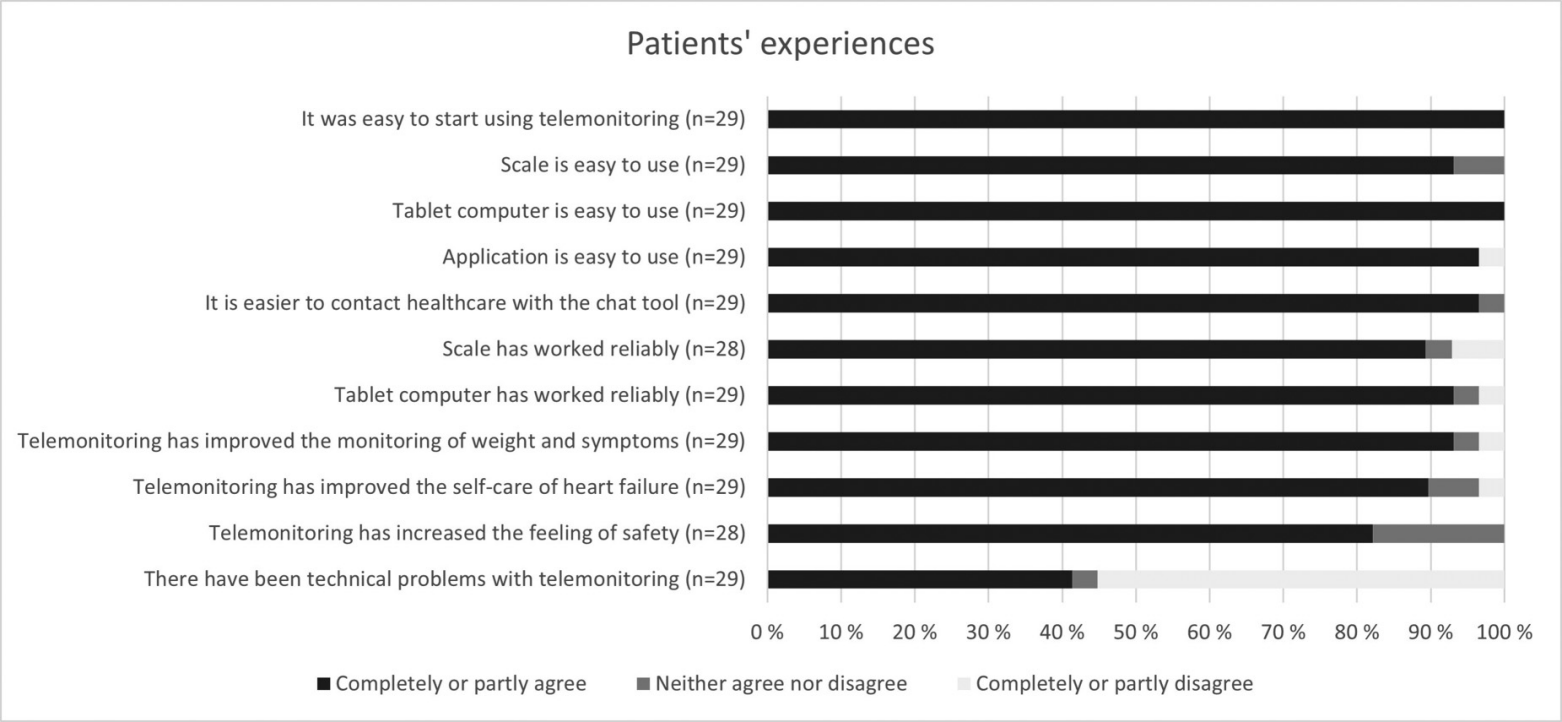
Patients’ experiences of the telemonitoring system by responses to the questionnaire.

The user experience questionnaire for patients assessed the feasibility of the telemonitoring system (Figure 3). On a Likert scale, patients answered that all parts of the telemonitoring system (scale, tablet computer, and application) are easy to use (93%, 100%, and 97% completely or partly agreed, respectively). Most patients found that the chat tool of the application made it easier to contact healthcare. The self-monitoring and self-care of heart failure were also improved with telemonitoring according to the answers. Almost half of the patients (41%) responded that there had been technical problems with telemonitoring. However, most of the patients considered that the scale and the tablet computer worked reliably (89% and 93% completely or partly agree, respectively).

Most patients answered that using the telemonitoring system usually took 0–5 min daily, with only one patient answering it took 5–10 min daily. Only 17 patients responded to the question on how likely it would be that they would recommend the telemonitoring system to a friend or a relative, but according to respondents, the net promoter score was 76.

### Qualitative data

#### Thematic analysis of the focus group interviews

**Figure 4.**
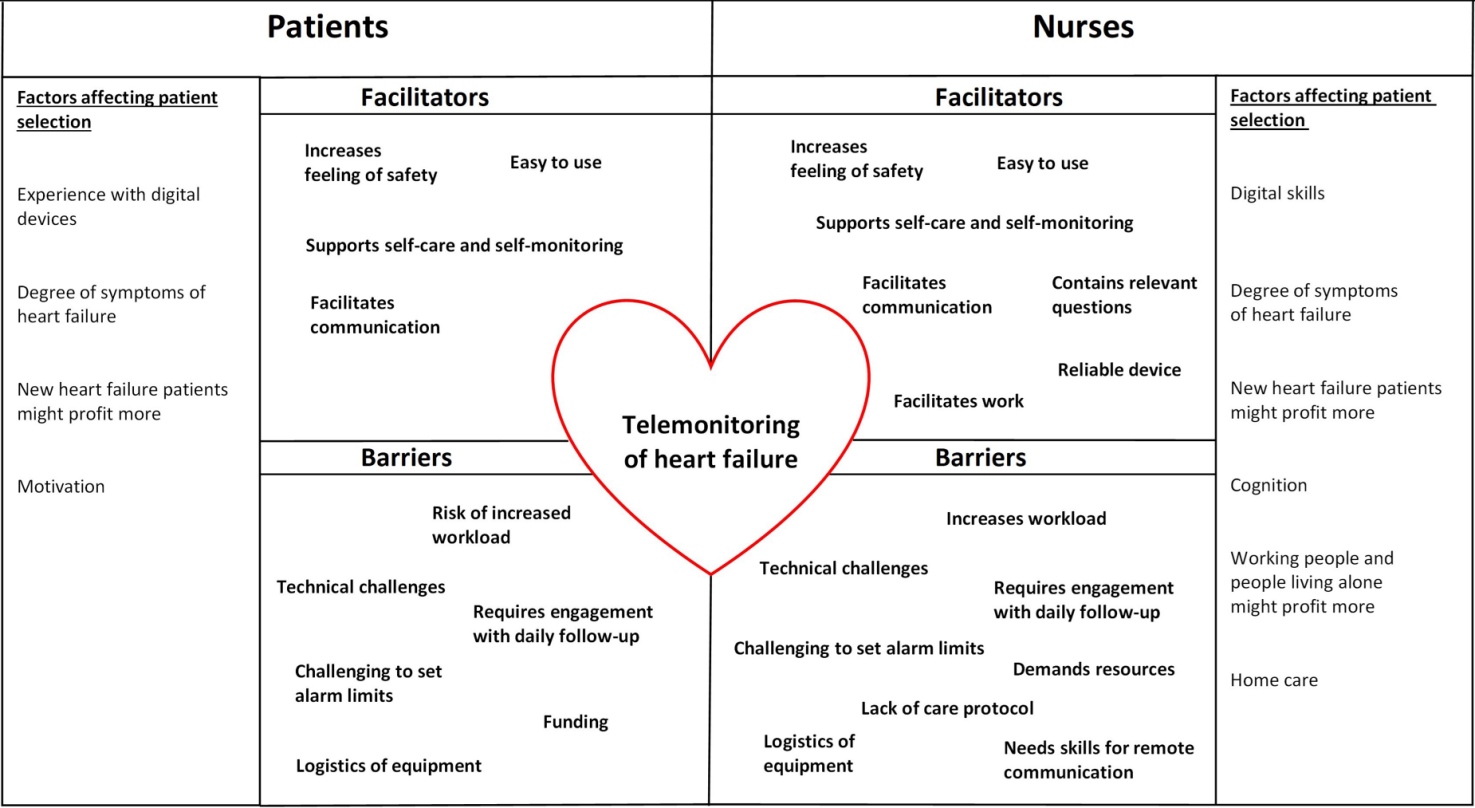
Themes including facilitators and barriers to telemonitoring and factors affecting patient selection arising from the focus group interviews.

**Figure 5.**
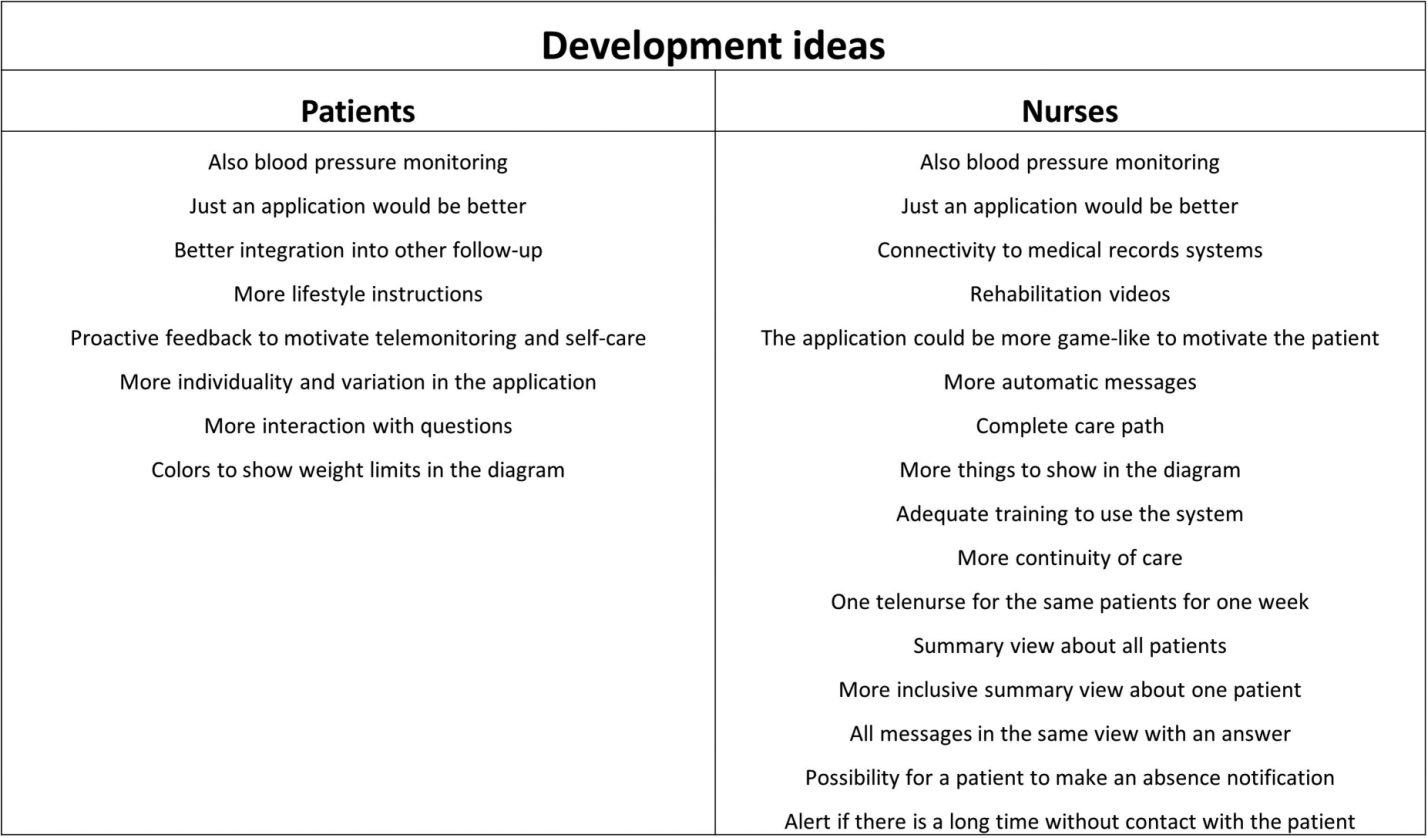
Development ideas for telemonitoring arising from the focus group interviews.

In the thematic analysis of the focus group interviews, we identified 10 themes from the patient interviews and 15 themes from the nurse interviews. We categorized all themes into facilitators and barriers in regard to telemonitoring. In addition, we identified factors affecting patient selection for telemonitoring and development ideas for telemonitoring that arose from the focus group interviews. The themes and factors affecting patient selection are shown in Figure 4, while the development ideas are shown in Figure 5. The themes with patients’ and nurses’ quotations are described below.

##### Facilitators of telemonitoring

###### Easy to use

Both the patients and nurses in the focus group interviews considered that it was easy to start using the telemonitoring system. Both the equipment and the application were simple and easy to use according to them. Some patients said that it became a routine to use the system.

*That kind of touch screen is easy and simple to use; it has no redundant buttons.* —Patient

*Quite fast it became a clear routine for me.* —Patient

*The application is very simple. Right away the first time you open it and get in, you know how to use it.* —Nurse

In addition, the layout of the application was assessed as being clear.

*I think the text and all is clear.* —Patient

###### Supports self-care and self-monitoring

The patients and nurses felt that the telemonitoring system supports and improves the self-care of patients. They also said that it supports the patients’ symptom monitoring and weight control.

*The encouragement to follow your own condition is essential.* —Patient

*It has affected weight control. … When you jump on the scale every morning, you get to know a little bit how to move and what to eat so the weight won't increase.* —Patient

*And then the fact that people learn how to treat themselves, the self-care increases, and the people focus on self-care in a different way.* —Nurse

Furthermore, the telemonitoring system helped the nurses with guiding the patients’ self-care of heart failure.

*It eases the burden since they practice at home with their device. It eases the guidance work.* —Nurse

###### Increases feeling of safety

Both the patients and nurses reported that telemonitoring increases the patients’ feeling of safety.

*In this system, there is exactly this kind of safety that there are professionals giving help right away when something worries you.* —Patient

*I would say that it has increased the overall feeling of safety.* —Patient

*The patients have been very excited that they can be part of this kind of system and get this service for free, so maybe it creates that kind of feeling of safety for them. —*Nurse

###### Facilitates communication

The chat tool of the application allows bidirectional messaging between patients and nurses, regardless of time. According to the answers in the focus group interviews, the tool offers an easy way to communicate between patients and nurses. It also lowers the patients’ threshold to contact nurses. The nurses said that it is easier for them to schedule their day to answer messages than to wait for phone calls. Both the patients and nurses considered this feature very important.

*You can contact nurses right away, if needed. That is probably the most important thing.* —Patient

*So, the threshold to write is much lower than to try to reach someone by phone.* —Patient

*So, it will become an easier way to contact patients.* —Nurse

In addition, nurses found it useful that the messages they sent left written advice for patients.

*The patient gets written advice from us, and it stays there. *—Nurse

###### Contains relevant questions

The nurses stated that the questions in the application of the telemonitoring system are relevant and describe those issues of the patient's condition that should be followed.

*And on the other hand, for all those questions, I think they work for most patients because false alarms don’t come from most of the patients.* —Nurse

*I think they are good questions and are relevant to describe those issues about the patient's condition that should be followed.* —Nurse

###### Facilitates work

According to the nurses, the telemonitoring system allows following patients without visits to the clinic and it makes it easier to see the results of their guidance to patients. It also can reduce phone calls to the clinic and give nurses more time for patients with problems.

*One nice benefit is that if I have advised a patient in some way, in a couple of days I can see how my guidance has worked from the telemonitoring system without contacting the patient.* —Nurse

*Earlier every contact has been by phone or face-to-face, so this is a kind of third dimension. I would think that it changes work so that I have more time to speak with those patients who have more grief.* —Nurse

The nurses indicated also that telemonitoring makes the work more variable and satisfying.

*It brought variability to my job duties.* —Nurse

*It brings satisfaction to my work when I have been able to influence somebody's health in a good way, hopefully.* —Nurse

###### Reliable device

Although there had been some technical challenges with the telemonitoring system, the nurses commented that generally the device had been reliable and the data transfer worked.

*I think that the telemonitoring system has worked pretty reliably and there haven’t been days when it hasn’t worked.* —Nurse

*But mainly the data transfer has worked well and the answers from patients have arrived.* —Nurse

##### Barriers to telemonitoring

###### Increases workload

The nurses felt that telemonitoring increases their workload, although it makes work easier. It takes time to guide a patient in the use of the telemonitoring system. The continuous follow-up might also cause extra work. Nurses reported that in the pilot program of telemonitoring, the minimum time it took was about 15 min per day, and half an hour per day was usually not enough.

*It makes work easier but has also brought more work because someone has to teach the application to the patient, hand over the equipment to the patient, and start the telemonitoring with the patient.* —Nurse

*If we wouldn’t normally follow patients continuously and it wouldn’t be our normal work, then it would be extra work.* —Nurse

One patient wondered similarly if there is a risk of increased workload for nurses caused by increased communication.

*Is there a risk in this system that someone sends a message right away when something feels a little bit different, and by doing so increases the nurses’ workload for such temporary disturbances in the body?* —Patient

###### Technical challenges

Both patients and nurses reported that there had been some technical problems with the telemonitoring system. Mostly, these cases were connection problems between the scale and tablet computer.

*Sometimes my scale and tablet computer lose their Bluetooth connection quite often.* —Patient

*There are some patients who don’t get a weight measurement from the scale to the tablet computer so they enter the measurement manually, and it's hard to say if the problem is with the batteries, with Bluetooth, with the scale, or why the measurement doesn’t come automatically.* —Nurse

The patients also indicated that technical challenges could weaken trust in telemonitoring.

*That kind of system should be reliable because when you can’t make contact the first time, you think what the problem is, is it because of this equipment, does anyone react to your contact in any way, and does this system work at all in the end?* —Patient

###### Requires engagement with daily follow-up

Daily follow-up can be distressing for the patients according to both the patients and nurses. This is true especially if nothing in particular has happened in a long time and the patients do not see the benefit to the follow-up.

*I feel a little bit distressed about the weight monitoring.* —Patient

*I often look at the diagram for a longer time and discover that nothing in particular has happened, so I made a decision not to measure my weight every morning.* —Patient

Both the patients and nurses said that feedback from the nurses is important in motivating the patients.

*And many patients probably leave telemonitoring because they have to commit to it so intensively every day. And especially if they don’t see any benefit and don’t get any feedback on how they are doing, what are the things that motivate them to use the telemonitoring system?* —Nurse

###### Challenging to set alarm limits

In the interviews, the nurses stated that it is challenging to set the right weight goal and limits in the application of the telemonitoring system.

*It is surprisingly hard to define the desirable weight for each patient at the appointment… And when adjusting the alarm limits, it is hard to say what is an acceptable weight loss and weight gain.* —Nurse

The challenge of setting the alarm limits can cause problems. In the interviews, the patients said that too rapid reactions to weight changes are annoying. The nurses also felt that unnecessary information on weight changes increases their workload.

*But the reaction to only one measurement, for me it is a little bit… I get annoyed and think how my weight is any of your business.* —Patient

*For example, there comes useless information on weight changes, which increases my workload* —Nurse

In addition, incorrect alarm limits can make it more difficult to know if the patient is feeling well according to nurses.

*It's dangerous if you don’t hear anything from the patient since you don’t know if it's because the patient is feeling well or because we have set incorrect alarm limits.* —Nurse

###### Logistics of equipment

Both the patients and nurses commented that it is difficult to carry the heavy equipment of this telemonitoring system.

*I think if you go somewhere, it is difficult to take the scale with you.* —Patient

*Patients complain that the briefcase (including the scale and the tablet computer) is heavy… and it is very thick, it isn’t easy to carry.* —Nurse

###### Funding

One patient wondered how willing society is to invest in this kind of system.

*Is society ready to invest in this?* —Patient

###### Demands resources

The nurses expressed a demand for a sufficient amount of personnel and a doctor to consult.

*I think that because we also have the phone to answer and we have to take care of the guidance clinic and other things, we need enough resources if the number of telemonitoring patients increases.* —Nurse

*Our heart failure doctors don’t have time to help us every time we need it.* —Nurse

###### Lack of care protocol

The nurses reported that at the beginning of the telemonitoring program, there was a lack of agreed practices and working protocols for professionals.

*And at the beginning, we didn’t have any agreed-on practice about what kind of alarms we make an intervention on and how we make an intervention.* —Nurse

*I don’t think there is any kind of process planned yet… there are still fundamental questions open, like what kinds of patients we take along.* —Nurse

In addition, the nurses wondered if they had duties that belonged to some other professionals.

*It doesn’t necessarily belong in our profession to pair devices and do other kinds of things we have to learn in this telemonitoring program. So maybe it is somebody else's job to do.* —Nurse

###### Needs skills for remote communication

The nurses felt that communication via the chat tool and by written messages requires new kinds of communication skills. Challenges with these skills and especially misspellings can lead to extra work.

*So, one has to think how I would like…, how I formulate this message.* —Nurse

*There are misspellings by patients, and it leads to unnecessary contacts.* —Nurse

##### Factors affecting patient selection

In the focus group interviews, both the patients and nurses reported factors affecting patient selection for telemonitoring. These issues were expressed when they were discussing the challenges of using the telemonitoring system and the things that affect the expansion of telemonitoring. The patients and nurses had mainly similar but also some diverse thoughts on these factors (Figure 4). They both pointed out digital skills, difficulty of heart failure, and new heart failure patients as important factors. One nurse thought that working people might benefit from telemonitoring, since they know how to use the system and the chat tool might ease contacting them during working hours. The nurses also wondered if people living alone would feel safer with telemonitoring and if the telemonitoring system would help monitoring patients in home care. Below are a few quotations about patient selection from the focus group interviews.

*That system brings you more if you have grief and problems, and things are not stable.* —Patient

*For some patients, using digital devices isn’t so typical.* —Nurse

*When there are new heart failure patients, they could benefit from the system to learn self-care and self-monitoring.* —Nurse

##### Development ideas

In the focus group interviews, the patients and nurses introduced many development ideas for the telemonitoring system (Figure 5). The ideas included both technical and practical improvements. The most common ideas were adding blood pressure monitoring to the telemonitoring system and the desire that the telemonitoring system would consist of just a smartphone application.

##### Responses to the open-ended questions of the questionnaire

The responses to the open questions of the questionnaire were mainly similar to the themes and answers arising from the focus group interviews. Most respondents felt that the telemonitoring system is good, easy to use, motivates the patient, and has made communication with nurses easier. Responses to development ideas included comments that daily questions in the application should be more open, blood pressure monitoring could be added to the system, and a smartphone application would be good. Most responses about technical difficulties were connection problems between the scale and the tablet computer.

## Discussion

### Principal results

In this mixed methods study, we examined the feasibility of the noninvasive heart failure telemonitoring system. The principal results are based on both quantitative and qualitative data of the study and the answers from all the user groups. Our primary findings are that the telemonitoring system is easy to use, supports the self-care and self-monitoring of heart failure, facilitates communication between the patients and nurses, and increases the patients’ feeling of safety. However, technical challenges with the system, the increased workload of nurses, and the patients’ engagement with continuous follow-up can be possible barriers to telemonitoring. We also identified factors affecting patient selection for telemonitoring and development ideas for telemonitoring.

### Comparison with prior work

#### Easy to use

In our study, the answers to both the questionnaire and discussions in the focus group interviews highlighted that it is easy to start and continue using the telemonitoring system. These findings are similar to other studies.^20,22,23,25^ The telemonitoring systems used in the previous studies have had similar features as the system in our study. The systems have included mobile applications for patients and web-based interfaces for professionals, and they have monitored different parameters including weight, blood pressure, and heart rate.^20,22,23,25^ It seems that at least these kinds of telemonitoring systems are easy to use.

In the UTAUT and UTAUT2 models by Venkatesh et al., ease of use (i.e. effort expectancy) is suggested to be an important factor especially for women, people of older age, and those with little prior experience of the system.^29,30^ This should be considered when developing heart failure telemonitoring systems since the majority of heart failure patients are elderly.^2^ However, an older age does not necessarily correlate with lower experience. In our study, approximately 60% of the patients were over 60 years old, but 89.7% of the patients had used digital devices daily before the study.

#### Supports self-care and self-monitoring and increases the feeling of safety

In our study, both the quantitative and qualitative data show that telemonitoring supports the self-care and self-monitoring of heart failure. These findings are in line with earlier studies.^18–20,25^ In a study by Ware et al. (2019), almost all (95.8%) patients agreed that the telemonitoring system is important for managing their heart failure. In the interviews, the most commonly mentioned benefit of the telemonitoring program was supporting patients in their ability to self-manage their heart failure.^20^ In other studies, patients have noted that telemonitoring promotes confidence in self-management,^18,25^ and there have been improvements in SCHFI (the self-care of heart failure index) maintenance and management scores.^19^

The patients in our study also reported an increased feeling of safety with telemonitoring in both the questionnaire and interview answers. In earlier studies, patients have similarly mentioned that telemonitoring has given them a sense of safety^18,25^ and peace of mind^18,20^ and has reduced anxiety.^26^

#### Facilitates communication

In the focus group interviews of our study, both patients and nurses indicated that the chat tool of the telemonitoring system offers an easy way to communicate. It lowers the threshold of contact because the patient does not need to try to contact nurses by phone. This can increase the number of contacts but can also reduce the amount of phone calls to healthcare. It is also easier for nurses to schedule their day to answer messages than to wait for phone calls. Both patients and nurses considered the chat tool one of the most important features of the telemonitoring system.

The telemonitoring systems in prior feasibility studies did not enable this kind of chat tool.^20,22–27^ Additionally, in previous studies, there was little data on how telemonitoring affects communication. In a study by Prescher et al. (2013), it was found that 52.6% of patients noticed an improvement in contact with their primary physician.^25^ In our study, 97% of patients who answered the questionnaire felt that it is easier to contact healthcare with the system's chat tool. The difference between these two studies might result from the facilitating effect of this tool in our study.

#### Technical challenges

One of the possible barriers to telemonitoring is the technical challenges of the system. In our study, 41% of patients experienced some technical problems with telemonitoring. According to the answers to the open-ended questions of the questionnaire and the focus group interviews, the technical problems were mostly connection problems between the scale and tablet computer. Similar Bluetooth connectivity problems have been reported in previous studies.^20,31^ Different telemonitoring systems also seem to display slightly varying technical issues according to earlier studies.^20,32^

Despite the technical challenges, the patients and nurses in our study and also in earlier studies stated that the telemonitoring systems are reliable, the quality of the systems is high, and the data transfer in the main works well.^20,24^ It seems that the technical problems with the systems in previous studies and in our study were so minor that they did not affect the experience of the reliability of the system in general. However, even though the technical challenges were mild, it is important to have easy and fast access to technical support. If not taken care of quickly and easily, technical problems can weaken trust in telemonitoring and thus lead to decreased satisfaction and adherence according to the answers in our study and previous studies.^18,20,32^

#### Increased workload

In the focus group interviews of our study, especially nurses, but also one patient, reported the risk of increasing the nurses’ workload. While telemonitoring can make work easier and more satisfying, patient guidance on using the telemonitoring system, continuous follow-up, and the easy chat tool can also increase the workload. The increased workload as a possible barrier to telemonitoring has also been raised in some previous studies. In those studies, professionals described an increased workload due to alarms, particularly inappropriate alarms.^26,33^ The difficulty of setting alarm limits for weight changes was reported also in our study.

Our findings in the thematic analysis of the focus group interviews indicated some factors that might help to manage the risk of increased workload. These include enough resources, a clear care protocol for telemonitoring, and adequate integration of telemonitoring with other follow-ups. The nurses in our study pointed out that telemonitoring demands resources due to the increased communication channels with patients. The nurses also stated that there should be a clear care protocol already at the beginning of the telemonitoring. In addition, they desire a separate technical assistant for technical issues. The development ideas by the participants of our study include the better integration of telemonitoring with other follow-ups and better connectivity of the telemonitoring with the medical records system. Some earlier studies have also shown the challenges of integrating telemonitoring into other care.^26,33^

#### Adherence and engagement with telemonitoring

In our study, the patients’ engagement with daily follow-up was one of the possible challenges to telemonitoring reported by both the patients and nurses. Daily follow-up can be distressing for patients, especially if there have not been any particular changes to the patient's condition for a longer time or if the patient does not see how he/she benefits from the follow-up.

In previous studies, the adherence of patients to telemonitoring has ranged from 53.3% to 98.5%.^6,14,20,22,34,35^ The differences in the adherence rates between studies might be due to the different types of telemonitoring systems used and the differences in population characteristics and follow-up times of the studies. The time that the telemonitoring has been used and the age of the patient seem to be predictors of adherence. In previous studies, adherence has been lower at the start of the telemonitoring, then increasing and peaking between 1 and 2 months after the start, but then gradually decreasing over time.^20,35^ The decrease over time has been seen especially with younger patients.^20^

High adherence to telemonitoring is important for achieving its possible benefits. In a study by Haynes et al. (2020), higher adherence to weight telemonitoring was associated with a decrease in the rates of death and hospitalization in the following week.^35^ According to the patients’ and nurses’ answers in our study, possible ways to improve engagement and adherence would be regular feedback from professionals and alternating or more interactive daily questions in the application of the telemonitoring system.

#### Patient selection

Correct patient selection for telemonitoring is essential for gaining the best possible benefits. The patients and nurses in our study described many patient-related factors that can affect how patients profit from telemonitoring. They both considered that patients with adequate digital skills, patients with more symptoms or challenges with heart failure, and new heart failure patients might profit more from telemonitoring.

Only a few studies have reported these factors earlier. In a qualitative study by Sivakumar et al. (2022), healthcare professionals described several factors that may affect the use of mobile apps by patients. These included the patients’ age, access to a smartphone or the Internet, familiarity with technology, physical and cognitive function, and motivation and engagement in telemonitoring and self-management.^36^ The digital skills, motivation, and cognitive skills of patients were also addressed in our study. The subgroup analyses of two earlier RCT studies show that patients who have more challenges with heart failure (higher NYHA-class, CRT-pacemaker), have higher adherence to weight measurement, or are socially isolated might benefit more in regard to reduced mortality and hospitalizations.^15,16^ These findings match the answers of the focus group interviews in our study.

However, there is no comprehensive data yet on which heart failure patients benefit most from telemonitoring systems. Further research and subgroup analyses with different heart failure patients are needed to better understand patient-related factors in this matter.

#### Development ideas

In the focus group interviews, the patients and nurses pointed out many development ideas for the system. These are very important to consider when developing the system and engaging and motivating the users within telemonitoring.

The development ideas from our participants include both technical and practical improvements. The most common ideas were adding blood pressure monitoring to the telemonitoring system and the desire that the telemonitoring system would consist of just a smartphone application. The patients would like to have an archive and diagram also for their blood pressure. The nurses, on the other hand, could use blood pressure to help with assessing the patient's condition and making medication dose changes. A simple smartphone application would ease use since the patient would not need to carry the heavy equipment everywhere. On the other hand, if only the application would be available, patients would have to have smartphones and other needed devices (e.g. scales) by themselves. This might reduce the reliability of the devices. Other themes the participants described included, for example, more motivating and individual features in the application, layout ideas for the application, more self-care instructions and videos, and more continuity of care.

A couple of studies have previously addressed patients’ and professionals’ suggestions for improvements.^27,32^ Buck et al. (2017) studied older adults’ perceptions of one telerehabilitation program where the mobile application was not connected to healthcare. They had similar development suggestions as in our study, including adding blood pressure monitoring and more self-care instructions to the system.^32^ The study by Bylappa et al. (2022) on a smartphone application reported some development ideas by healthcare professionals to improve functionality. The ideas included, for example, an alarm feature and chat tool within the app.^27^ These features were already included in the telemonitoring system in our study.

### Limitations and strengths

One limitation of this study is the small sample of participants. At the time of the study, the telemonitoring system was at the pilot stage and had been in use for about a year. There were 47 patients participating in the pilot program at the Heart Hospital unit in Tampere. We wanted to examine the feasibility of this particular telemonitoring system. As a result, we sent the questionnaire and informed consent form to those 47 patients. Even though the response rate to the questionnaire was reasonably high (61.7%), the final number of participants was small (29 patients). Therefore, there might be some selection bias in our study population. The patients enrolled in the pilot program and consequently in our study might be more engaged and interested in telemonitoring compared to patients who declined to participate. Furthermore, male patients were overrepresented in our sample compared to the heart failure population in general. This is consistent with other studies of heart failure, where women are easily under-represented.^37^ The mean age of patients in our study was 61 years, which is lower than the mean age of heart failure patients generally.^2,4^ Still, there were many elderly patients in our sample, and most patients were over 60 years old.

The differences in our study population compared to the heart failure population generally might be due to the recruitment of patients from the Heart Hospital unit in Tampere, which treats a high proportion of severely ill heart failure patients and young heart failure patients in Finland. The nurses who participated in our study were also mostly from the Heart Hospital units. Consequently, they might be more interested and eager to try new ways to treat heart failure patients compared to professionals in some other healthcare units. However, we also had two nurses from primary health care in the focus group interviews to reduce this possible bias.

The other limitation of our study is the deficiency of the theoretical model behind the questions of the questionnaire and the frames of the interviews. The questions and the frames were planned by the research group to study the particular telemonitoring system used in the pilot program. The objective was to obtain information about user experiences and the feasibility of this system. Nevertheless, some theoretical models, for example, UTAUT or UTAUT2, might have given us more information on the technology acceptance of the system.^29,30^

Even though the UTAUT or UTAUT2 models were not used in our study, our findings can be connected with some constructs of the models.^29,30^ In our study, effort expectancy (i.e. how easy it is to use the system) was well examined. Performance expectancy (i.e. how telemonitoring is perceived to provide benefits) was studied for parts of support for self-care and self-monitoring, the increased feeling of safety, and the facilitating effect of the telemonitoring system on communication. The participants in our study also mentioned some factors of facilitating conditions (technical support and lack of care protocol), habit (engagement with daily follow-up, ease of use), and price value (funding). Our findings do not include any factors from the constructs of social influence (i.e. influence of perceptions of important others) or hedonic motivation (i.e. enjoyment of using telemonitoring). This might be due to the nature of the telemonitoring system used in our study, but the deficiency of the theoretical models behind our methods may also be a factor.

The strength of this study is the mixed methods design, wherein we obtained information about the feasibility by both quantitative and qualitative methods. The answers to the questionnaire gave us a general view on end-user experiences and the focus group interviews deepened and expanded our understanding of those experiences. In addition, we asked for experiences from both patients and nurses using the telemonitoring system. In this way, we got a more comprehensive view of the feasibility of the system than just by asking only one of these user groups.

### Implications

The telemonitoring system in our study included a bidirectional chat tool for patients and healthcare professionals. This made communication easier, and it was considered to be one of the most important features of the system. Based on these findings, this kind of chat tool in telemonitoring seems useful. In future studies, it would be useful to assess the effect of telemonitoring on communication between patients and healthcare professionals in more detail.

Our findings also introduced some development ideas for the noninvasive telemonitoring of heart failure. Quick and easy access to technical support for patients is important in preventing decreased trust in telemonitoring caused by technical challenges. Regular feedback from professionals and more interactive questions might increase patients’ adherence and engagement with telemonitoring and continuous follow-up. In addition, a clear care protocol, the integration of telemonitoring into other follow-ups, and sufficient resources are important for controlling the workload of professionals and engaging them with telemonitoring. Other development ideas reported by our study participants include adding blood pressure monitoring to the telemonitoring system and the possibility of having just a simple smartphone application for telemonitoring. All these ideas have been taken into account within the development of the studied telemonitoring system, and they can also be considered when developing other noninvasive telemonitoring systems. In the future, artificial intelligence might help with many of these issues, especially with issues concerning communication, interactivity, and the workload of professionals.

Correct patient selection is important to gain the best possible benefits from the telemonitoring of heart failure. The patients and nurses in our study felt that patients with adequate digital skills, patients with more symptoms or challenges with heart failure, and new heart failure patients might profit more from telemonitoring. However, there is still little data on which patients would benefit most from the telemonitoring of heart failure. Further research is needed to obtain more information on the effectiveness of noninvasive telemonitoring in different patient groups.

We would also recommend further research on the feasibility of heart failure telemonitoring using the models UTAUT or UTAUT2. These models would give more information on all constructs affecting the technology acceptance of telemonitoring systems.

## Conclusions

The noninvasive heart failure telemonitoring system used in the pilot program is feasible. It is easy to use, supports the self-care and self-monitoring of heart failure, facilitates communication between the patients and nurses, and increases the patients’ feeling of safety. Technical challenges with the system, the increased workload of nurses, and the patients’ engagement with continuous follow-up can be possible barriers to telemonitoring. These factors should be considered when developing and expanding the noninvasive telemonitoring of heart failure in the future.

## Supplemental Material

sj-docx-1-dhj-10.1177_20552076241272633 - Supplemental material for Feasibility of a noninvasive heart failure telemonitoring system: A mixed methods study Supplemental material, sj-docx-1-dhj-10.1177_20552076241272633 for Feasibility of a noninvasive heart failure telemonitoring system: A mixed methods study by Teemu Ekola, Vesa Virtanen and Tuomas H Koskela in DIGITAL HEALTH

sj-docx-2-dhj-10.1177_20552076241272633 - Supplemental material for Feasibility of a noninvasive heart failure telemonitoring system: A mixed methods study Supplemental material, sj-docx-2-dhj-10.1177_20552076241272633 for Feasibility of a noninvasive heart failure telemonitoring system: A mixed methods study by Teemu Ekola, Vesa Virtanen and Tuomas H Koskela in DIGITAL HEALTH

sj-docx-3-dhj-10.1177_20552076241272633 - Supplemental material for Feasibility of a noninvasive heart failure telemonitoring system: A mixed methods study Supplemental material, sj-docx-3-dhj-10.1177_20552076241272633 for Feasibility of a noninvasive heart failure telemonitoring system: A mixed methods study by Teemu Ekola, Vesa Virtanen and Tuomas H Koskela in DIGITAL HEALTH

sj-docx-4-dhj-10.1177_20552076241272633 - Supplemental material for Feasibility of a noninvasive heart failure telemonitoring system: A mixed methods study Supplemental material, sj-docx-4-dhj-10.1177_20552076241272633 for Feasibility of a noninvasive heart failure telemonitoring system: A mixed methods study by Teemu Ekola, Vesa Virtanen and Tuomas H Koskela in DIGITAL HEALTH

sj-docx-5-dhj-10.1177_20552076241272633 - Supplemental material for Feasibility of a noninvasive heart failure telemonitoring system: A mixed methods study Supplemental material, sj-docx-5-dhj-10.1177_20552076241272633 for Feasibility of a noninvasive heart failure telemonitoring system: A mixed methods study by Teemu Ekola, Vesa Virtanen and Tuomas H Koskela in DIGITAL HEALTH

sj-docx-6-dhj-10.1177_20552076241272633 - Supplemental material for Feasibility of a noninvasive heart failure telemonitoring system: A mixed methods study Supplemental material, sj-docx-6-dhj-10.1177_20552076241272633 for Feasibility of a noninvasive heart failure telemonitoring system: A mixed methods study by Teemu Ekola, Vesa Virtanen and Tuomas H Koskela in DIGITAL HEALTH
