# Supplementary material for: Feasibility of a noninvasive heart failure telemonitoring system: A mixed methods study
Source: Digit Health. 2024 Sep 12;10:20552076241272633. doi: 10.1177/20552076241272633 (PMC11406595; doi:10.1177/20552076241272633)
Supplement: sj-docx-1-dhj-10.1177_20552076241272633 - Supplemental material for Feasibility of a noninvasive heart failure telemonitoring system: A mixed methods study [file sj-docx-1-dhj-10.1177_20552076241272633.docx]

**Appendix 1. Images of the patient application and the user interface for nurses**

The images are from power point presentation about Telemonitoring of heart failure patients (2020) by Heart Hospital Development Director Janne Hulkkonen. Janne Hulkkonen has given us the permission to use the images.


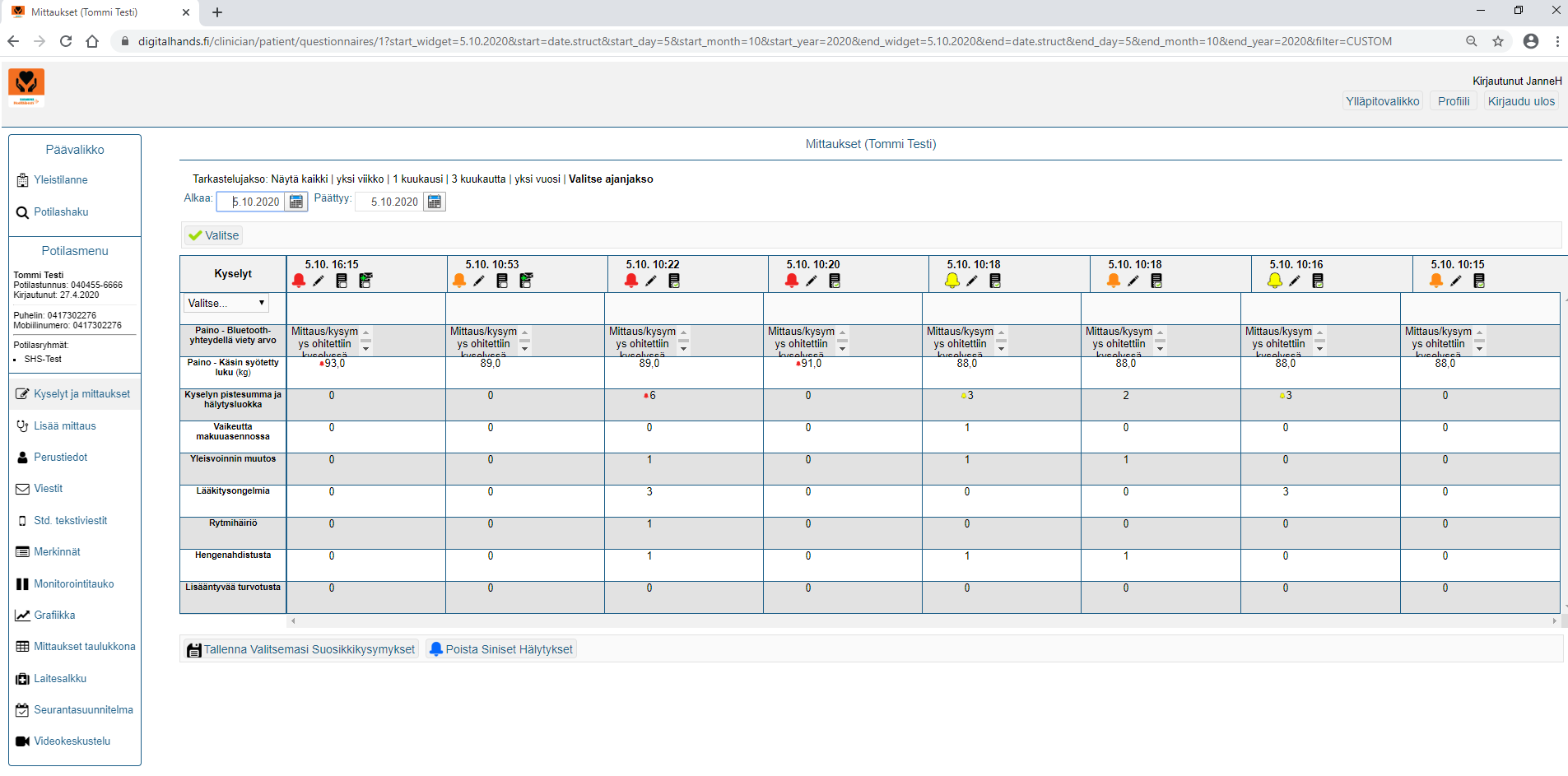

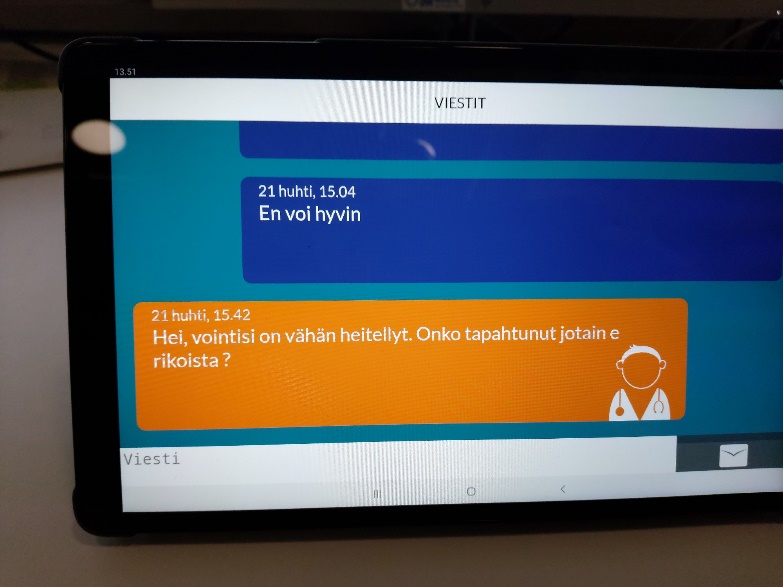

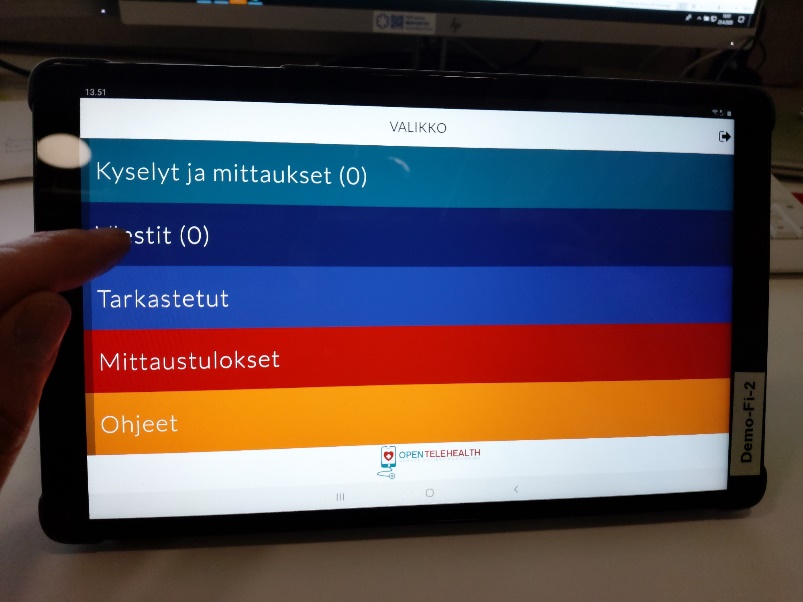


Image 3. User interface for nurses.

Image 2. Chat tool of the patient application.

Image 1. Menu of the patient application.
