# Supplementary material for: Feasibility of a noninvasive heart failure telemonitoring system: A mixed methods study
Source: Digit Health. 2024 Sep 12;10:20552076241272633. doi: 10.1177/20552076241272633 (PMC11406595; doi:10.1177/20552076241272633)
Supplement: sj-docx-2-dhj-10.1177_20552076241272633 - Supplemental material for Feasibility of a noninvasive heart failure telemonitoring system: A mixed methods study [file sj-docx-2-dhj-10.1177_20552076241272633.docx]

## Appendix 2. The questionnaire for patients.

1. My age ________ years

2. My gender 1) female

2) male

3. My height ________ cm

4. My weight ________ kg

5. I have had heart failure _______ years

6. I have been diagnosed with following cardiovascular diseases (you can choose multiple answers):

1. coronary artery disease
2. hypertension
3. valvular heart disease (e.g., aortic stenosis or mitral regurgitation)
4. atrial arrhythmia
5. cardiomyopathy (cardiomyopathy, infiltrative cardiomyopathy, or infectious myocarditis)
6. other, what? _____________________________

7. Treatments I have had for my heart disease (**circle** **all** alternatives concerning you)

1. PCI (percutaneous coronary intervention)
2. pacemaker
3. medication
4. other: ___________________________

In the following questions, **circle** the best alternative for you:

8. I have used digital devices (e.g., computer, tablet computer, mobile phone) earlier

| daily | weekly | monthly | less often than monthly | never |
| --- | --- | --- | --- | --- |
| 1 | 2 | 3 | 4 | 5 |

In the following questions, **circle** the best alternative for you:

| **9. Scale** | completely agree | partly agree | neither agree nor disagree | partly disagree | completely disagree |
| --- | --- | --- | --- | --- | --- |
| It was easy to start using the scale | 1 | 2 | 3 | 4 | 5 |
| There are adequate written instructions for using the scale | 1 | 2 | 3 | 4 | 5 |
| It is easy to step on the scale and get a weight measurement (easy to use the scale) | 1 | 2 | 3 | 4 | 5 |
| The weight measurement transfers automatically from the scale to the tablet computer | 1 | 2 | 3 | 4 | 5 |
| The scale has worked reliably | 1 | 2 | 3 | 4 | 5 |

| **10. Tablet computer (later tablet)** | completely agree | partly agree | neither agree nor disagree | partly disagree | completely disagree |
| --- | --- | --- | --- | --- | --- |
| It was easy to start using the tablet | 1 | 2 | 3 | 4 | 5 |
| There are adequate written instructions for using the scale | 1 | 2 | 3 | 4 | 5 |
| It is easy to switch the tablet on and off (easy to use the tablet) | 1 | 2 | 3 | 4 | 5 |
| The tablet computer has worked reliably | 1 | 2 | 3 | 4 | 5 |
| **11. “Digital Hands,” the telemonitoring application on the tablet** | completely agree | partly agree | neither agree nor disagree | partly disagree | completely disagree |
| It is easy to log in to the application | 1 | 2 | 3 | 4 | 5 |
| It is easy to use the application | 1 | 2 | 3 | 4 | 5 |
| The layout of the application is suitable for telemonitoring | 1 | 2 | 3 | 4 | 5 |

| **12. Application’s questions concerning the health condition** | completely agree | partly agree | neither agree nor disagree | partly disagree | completely disagree |
| --- | --- | --- | --- | --- | --- |
| It is easy to answer the questions | 1 | 2 | 3 | 4 | 5 |
| The questions are related to heart failure and my condition | 1 | 2 | 3 | 4 | 5 |
| It is easy to move from one question to another | 1 | 2 | 3 | 4 | 5 |
| How long does it take on average to answer the daily questions and step on the scale? | | | | | |
| expressed in minutes | 0–5 min | 5–10 min | 10–15 min | 15–20 min | over 20 min |

| **13. Automatic feedback messages, measurement results, and chat tool in the application** | completely agree | partly agree | neither agree nor disagree | partly disagree | completely disagree |
| --- | --- | --- | --- | --- | --- |
| Automatic feedback messages after answering the questions and measuring weight are adequate and suitable | 1 | 2 | 3 | 4 | 5 |
| It is easy to search and scan the measurement results afterward | 1 | 2 | 3 | 4 | 5 |
| It is easier to contact healthcare with the chat tool | 1 | 2 | 3 | 4 | 5 |
| It is easy to use the chat tool | 1 | 2 | 3 | 4 | 5 |

| **14. The guidance part of the application** | completely agree | partly agree | neither agree nor disagree | partly disagree | completely disagree |
| --- | --- | --- | --- | --- | --- |
| The guidance concerning heart failure includes enough information about the self-care of heart failure | 1 | 2 | 3 | 4 | 5 |
| The substance within the guidance is easy to open | 1 | 2 | 3 | 4 | 5 |

| **15. Technical problems** | completely agree | partly agree | neither agree nor disagree | partly disagree | completely disagree |
| --- | --- | --- | --- | --- | --- |
| There have been disturbing technical problems with the tablet computer and the “Digital Hands” application (telemonitoring application) | 1 | 2 | 3 | 4 | 5 |

What kind of technical problems have you noticed?

____________________________________________________________________________________________________________________________________________________________________________________________________________________________________________________

**16. Recommendation (NPS)**

How likely is it that you would recommend the telemonitoring system to a friend or a relative on a scale from 0 to 10?

(very unlikely) 0 — 1 — 2 — 3 — 4 — 5 — 6 — 7 — 8 — 9 — 10 (very likely)

| **17. Telemonitoring in general** | completely agree | partly agree | neither agree nor disagree | partly disagree | completely disagree |
| --- | --- | --- | --- | --- | --- |
| Telemonitoring has improved the monitoring of my weight and symptoms | 1 | 2 | 3 | 4 | 5 |
| Telemonitoring has improved the self-care of heart failure | 1 | 2 | 3 | 4 | 5 |
| Telemonitoring has increased my feeling of safety | 1 | 2 | 3 | 4 | 5 |
| Telemonitoring has improved my quality of life | 1 | 2 | 3 | 4 | 5 |

Free comments about the telemonitoring system or telemonitoring in general

_________________________________________________________________________________________________________________________________________________________________________________________________________________________________________________________

Possible development ideas

_________________________________________________________________________________________________________________________________________________________________________________________________________________________________________________________

Thank you for answering the questionnaire!
