# Supplementary material for: Feasibility of a noninvasive heart failure telemonitoring system: A mixed methods study
Source: Digit Health. 2024 Sep 12;10:20552076241272633. doi: 10.1177/20552076241272633 (PMC11406595; doi:10.1177/20552076241272633)
Supplement: sj-docx-4-dhj-10.1177_20552076241272633 - Supplemental material for Feasibility of a noninvasive heart failure telemonitoring system: A mixed methods study [file sj-docx-4-dhj-10.1177_20552076241272633.docx]

## Appendix 4. The framework of semi-structured focus group interview for nurses.

Icebreaker: How does it feel to attend this kind of interview?

**General topics**

- How has the follow-up for heart failure patients changed with telemonitoring?
  - what has been the effect on your work?
  - has it changed your working practices?
- What benefits have there been with the telemonitoring of heart failure?
  - concerning self-care and self-monitoring?
  - concerning communication with patients?
  - what are the best features of the telemonitoring system?
- What kinds of challenges have there been with the telemonitoring of heart failure?
  - how much time does it take?
  - have there been any technical challenges?
  - are there any barriers to using the telemonitoring system; what limits the use of telemonitoring?

**More detailed topics**

- Guiding the use of the telemonitoring system for the patient
  - facilitating factors
  - challenges
  - development ideas
- The technical operability of the telemonitoring system (e.g., web-based user interface, the transfer of patient’s measurements and answers to the user interface)
  - user-friendliness
  - problems
  - development ideas
- The layout of the user interface of the telemonitoring system (clearness, color, and size of the content)
- The content/parts of the user interface for the nurses: questions concerning patient’s condition, chat tool, general view, patient view, team notes, adding and editing patient information
  - good things about different parts
  - challenges with different parts
  - development ideas for different parts
  - what parts have you noticed/used? What parts have you not noticed/used?
  - what kind of matters are suitable for the communication tool?
- How would you like the telemonitoring system to be improved?
  - concrete development ideas
- Is there something else you would like to discuss concerning heart failure telemonitoring?
  - possible issues that have been addressed in answers to the questionnaire
