# Supplementary material for: Feasibility of a noninvasive heart failure telemonitoring system: A mixed methods study
Source: Digit Health. 2024 Sep 12;10:20552076241272633. doi: 10.1177/20552076241272633 (PMC11406595; doi:10.1177/20552076241272633)

**Appendix 6. Consolidated criteria for reporting qualitative studies (COREQ): 32-item checklist.**

**Checklist copied from study** Allison Tong, Peter Sainsbury, Jonathan Craig, Consolidated criteria for reporting qualitative research (COREQ): a 32-item checklist for interviews and focus groups, International Journal for Quality in Health Care, Volume 19, Issue 6, December 2007, Pages 349–357


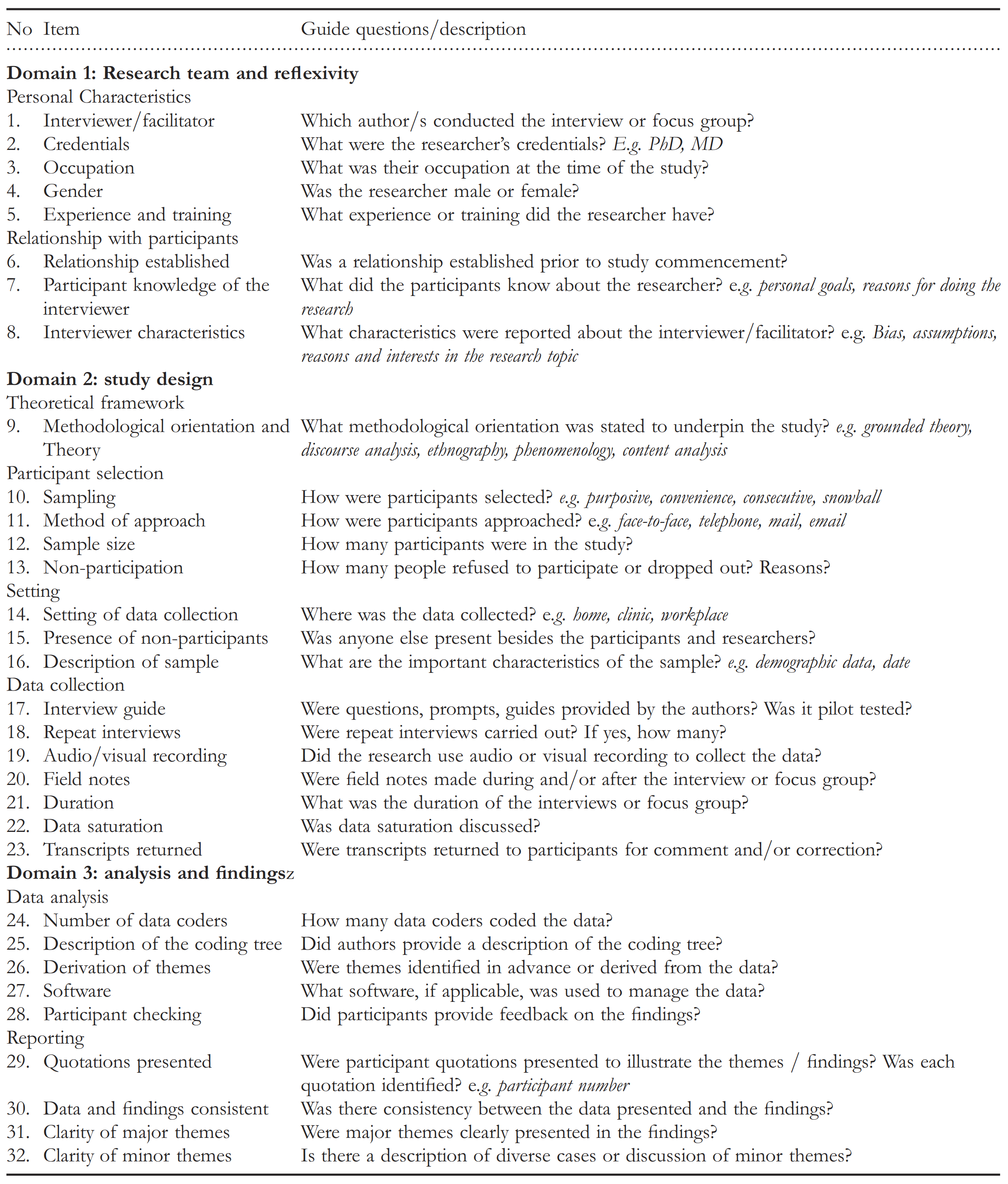

Supplement: sj-docx-6-dhj-10.1177_20552076241272633 - Supplemental material for Feasibility of a noninvasive heart failure telemonitoring system: A mixed methods study [file sj-docx-6-dhj-10.1177_20552076241272633.docx]
